# Perinatal Transmission of Yellow Fever, Brazil, 2009

**DOI:** 10.3201/eid1709.110242

**Published:** 2011-09

**Authors:** Maria Regina Bentlin, Ricardo Augusto Monteiro de Barros Almeida, Kunie Iabuki Rabello Coelho, Ana Freitas Ribeiro, Melissa Mascheratti Siciliano, Akemi Suzuki, Carlos Magno Castelo Branco Fortaleza

**Affiliations:** Authors affiliations: Universidade Estadual Paulista, Botucatu, Brazil (M.R. Bentlin, R.A.M. de Barros Almeida, K.I.R. Coelho, C.M.C.B. Fortaleza);; Centro de Vigilância Epidemiológica “Alexandre Vranjac,” São Paulo, Brazil (A.F. Ribeiro, M.M. Siciliano);; Instituto Adolfo Lutz Public Health Laboratory, São Paulo (A. Suzuki)

**Keywords:** viruses, yellow fever, vertical infectious disease transmission, perinatal transmission, infectious disease outbreaks, Brazil, letter

**To the Editor:** Although urban cases of yellow fever have not been reported in Brazil since 1942, sylvatic yellow fever is still endemic to the northern and middle-western states. In the past decade, the endemic area has spread southward and eastward, approaching most populated states (*1*). In 2009, there was an outbreak of sylvatic yellow fever in São Paulo State that caused 28 cases and 11 deaths. In the affected area, there had been no reports of yellow fever since the 1930s (*2*). In the outbreak setting, a case of perinatal yellow fever transmission was diagnosed.

The mother was a 30-year-old woman exposed to yellow fever in late pregnancy in a sylvatic area near Piraju (23°11′44′′S, 49°22′54′′W), a city 100 km from Botucatu. The patient had not received yellow fever vaccine and had not traveled to yellow fever–endemic regions in the previous months. The exposure to yellow fever occurred during regular walks in a sylvatic area, a habit that continued until late pregnancy. She had fever, headache, and jaundice on March 14, 2009. Three days later, on March 17, she delivered a female infant through vaginal partum in a hospital in her hometown.

The mother´s symptoms were mild. She was admitted to Botucatu Medical School Hospital 7 days after delivery; she had fever, jaundice, and conjunctival suffusion. Liver enzymes were elevated (aspartate aminotransferase [AST] 246 U/L, alanine transaminase [ALT] 324 U/L, γ-glutamyl transpeptidase 221 U/L, and alkaline phosphatase 338 U/L). She was mildly anemic (hemoglobin level 10.2 g/dL), but leukocyte and platelet counts were within reference ranges. There were no other laboratory abnormalities. She was discharged after 7 days with complete recovery.

The infant girl was born asymptomatic on March 17, with a birthweight of 3,800 g and Apgar scores of 9–10. She was discharged from the hospital after 2 days of exclusive breast-feeding. On the third day of life, she had fever and cyanosis and was readmitted to the local hospital with suspected pneumonia. She received antimicrobial drugs but showed no improvement. On the 8th day of life, she had hematemesis, melena, bleeding at venipuncture sites, hypoglycemia, and oliguria.

The newborn was transferred to the Neonatal Intensive Care Unit at Botucatu Medical School Hospital. At admission, she had hypotension, tachycardia, cutaneous paleness, jaundice, hepatomegaly, and melena. The initial diagnostic hypothesis was congenital or hospital-acquired sepsis, but the mother´s diagnosis prompted doctors to investigate possible yellow fever. The infant was intubated for ventilatory support and received volume expansion, vasoactive amines, antimicrobial drugs, blood components (erythrocytes, platelets, fresh frozen plasma, cryoprecipitate), and drugs to control bleeding. Liver enzymes values were initially high (AST 4,072 U/L, ALT 1,420 U/L) but fell abruptly within 3 days (AST 150 U/L, ALT 114 U/L).

Despite the therapy, the newborn experienced liver and renal failure, disseminated intravascular coagulation, seizures, and finally coma. She died on the 12th day of life (4th day of hospitalization in the neonatal intensive care unit). Autopsy specimens showed massive liver necrosis, pulmonary hemorrhage, and acute tubular necrosis (Figure).

**Figure:**
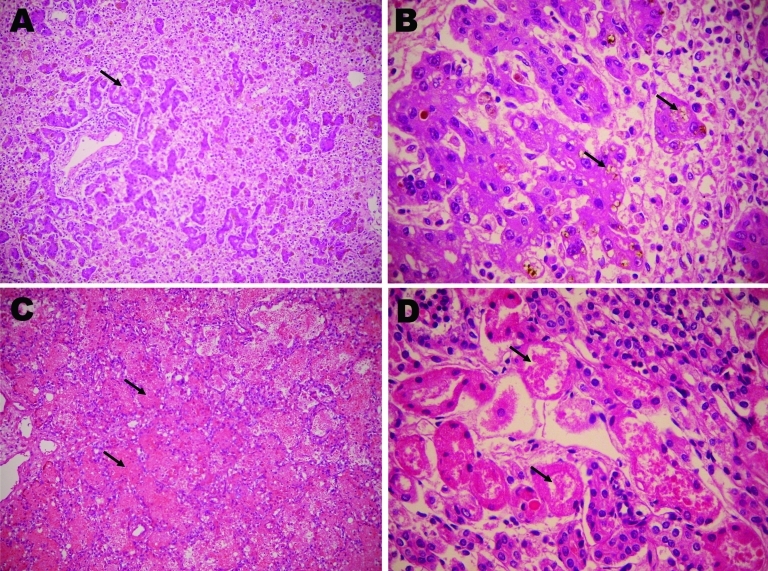
Microscopy findings of autopsy specimens from a 12-day-old girl with yellow fever, Brazil, 2009. A) Massive liver necrosis, with proliferation of ductular-like structures around portal tracts (arrow); B) hepatocytes with microvesicular fatty changes (arrows); C) intra-alveolar lung hemorrhage (arrows); D) renal tissue showing acute tubular necrosis (arrows). Panels A and C, original magnification ×200; panels B and D, original magnification ×400.

The mother had serologic tests (immunoglobulin M antibody capture ELISA) done on the 11th day of disease. Test results were positive for yellow fever and negative for dengue fever. The newborn had similar results from serum samples collected 5 days after onset of symptoms with confirmation by reverse transcription PCR (RT-PCR). RT-PCR was performed as described by Deubel et al. (*3*). Nucleotide sequencing showed a wild yellow fever virus belonging type I of South American genotype 1E, according to the classification proposed by Vasconcelos et al. (*4*). RT-PCR performed with samples of the mother´s serum did not amplify yellow fever virus sequences. However, the serum was collected on the 11th day post symptoms when the sensitivity of the test is low.

The vertical transmission of arboviruses has been documented. Pouliot et al. reviewed direct and indirect evidence for vertical transmission of dengue virus (*5*). Vertical transmission has also been reported for West Nile encephalitis and western equine encephalitis (*6*). This is not the case for yellow fever. Reports of yellow fever during pregnancy are scarce (*7*), and we found none that describe vertical transmission to newborns. However, vaccine virus was isolated from asymptomatic newborns from pregnant women who were inadvertently administered 17D vaccines (*8*). Still, mother-to-child transmission in late pregnancy or during delivery is a likely explanation for this newborn infection. The possibility of acquiring the virus through a mosquito bite is unlikely because urban cases of yellow fever have not occurred in Brazil in the past 50 years and were not reported in the present outbreak.

The timing of the newborn’s symptoms also argues against the possibility of transmission from a mosquito. We cannot rule out transmission through breast-feeding, which has been reported for the yellow fever vaccine virus (*9*). However, this would presuppose a short incubation period. In conclusion, this case points out to the possibility of vertical transmission of yellow fever. We imagine that similar cases can occur in yellow fever–endemic settings and may be identified by improved surveillance.

## References

[1] Vasconcelos PF. Yellow fever in Brazil: thoughts and hypothesis on the emergence in previously free areas. Rev Saude Publica. 2010;44:1144–9. 10.1590/S0034-89102010005000046 21109907

[2] Centro de Vigilância Epidemiológica. Febre amarela silvestre, Estado de São Paulo, 2009 [cited 2011 Jan 21]. ftp://ftp.cve.saude.sp.gov.br/doc_tec/ZOO/Boletim_FASP171209.pdf

[3] Deubel V, Huerre M, Cathomas G, Drouet MT, Wuscher N, Le Guenno B, Molecular detection and characterization of yellow fever virus in blood and liver specimens of a non-vaccinated fatal human case. J Med Virol. 1997;53:212–7. 10.1002/(SICI)1096-9071(199711)53:3<212::AID-JMV5>3.0.CO;2-B 9365884

[4] Vasconcelos PF, Bryant JE, da Rosa TP, Tesh RB, Rodrigues SG, Barrett AD. Genetic divergence and dispersal of yellow fever virus, Brazil. Emerg Infect Dis. 2004;10:1578–84. 15498159 10.3201/eid1009.040197 PMC3320275

[5] Pouliot SH, Xiong X, Harville E, Paz-Soldan V, Tomashek KM, Breart G, Maternal dengue and pregnancy outcomes: a systematic review. Obstet Gynecol Surv. 2010;65:107–18. 20100360 10.1097/OGX.0b013e3181cb8fbc

[6] Tsai TF. Congenital arboviral infections: something new, something old. Pediatrics. 2006;117:936–9. 10.1542/peds.2005-2729 16510678

[7] Sicé A, Rodallec C. Manifestations hémorragiques de: la fièvre jaune (typhus amaril). Répercussions de l’infection maternelle sur l’organisme foetal. Bull Soc Pathol Exot. 1940;33:66–9.

[8] Tsai TF, Paul R, Lynberg MC, Letson GW. Congenital yellow fever virus infection after immunization in pregnancy. J Infect Dis. 1993;168:1520–3. 10.1093/infdis/168.6.1520 8245539

[9] Centers for Disease Control and Prevention. Transmission of yellow fever vaccine virus through breast-feeding—Brazil, 2009. MMWR Morb Mortal Wkly Rep. 2010;59:130–2. 20150888

